# Supplementary material for: Tetraspanin-8 sequesters syntaxin-2 to control biphasic release propensity of mucin granules
Source: Nat Commun. 2023 Jun 22;14:3710. doi: 10.1038/s41467-023-39277-9 (PMC10287693; doi:10.1038/s41467-023-39277-9)
Supplement: Supplementary file 1 — Supplementary Information Data [file 41467_2023_39277_MOESM1_ESM.pdf]

Biphasic release propensity of mucin granules is supervised by tetraspanin-8 by sequestering syntaxin-2

Wojnacki José[1], Lujan Agustín[1], Brouwers Nathalie[1], Aranda-Vallejo Carla[1,2], Bigliani Gonzalo[1], Pena Rodriguez Maria[1], Foresti Ombretta[1] & Malhotra Vivek[1,2,3*]

[1] Centre for Genomic Regulation (CRG), The Barcelona Institute for Science and Technology, Barcelona, Spain.

[2] Universitat Pompeu Fabra (UPF), Barcelona 08002, Spain.

[3] ICREA, Barcelona 08010, Spain.

*Corresponding author and email: vivek.malhotra@crg.eu

Supplementary Methods

Supplementary Table 1. Sequences of the RNA interference (RNAi) used.

RNAi sequence 5' to 3'	Target gene	Purpose	Target cell
CUAGACAAGCUCUCAAUGAAAUCGA	STX2	KD	HT29-N2
GCUAUGUUGAAGGUAUCACUUGUCT	STX2	KD	HT29-N2

Supplementary Table 2. List of guide RNA (sgRNA) sequences.

Guide sequence	Target gene	Purpose	Target cell
cggcagtgctggtcagtgca	MUC5AC (c-terminus)	KI	HT29-N2
tgttcccgatctggcaatac	TSPAN8 (c-terminus)	KI and KO	HT29-N2
acccagaacaagaagttga	TSPAN8	KO	HT29-N2
caatatgtccacagcaacgt	TSPAN8	KO	HT29-N2
catggatgatttcttccatc	STX2	KO	HT29-N2
cattctttctgcaccaaacc	STX2	KO	HT29-N2
ctgtttcgggagcggagcaa	STX2	KO	HT29-N2

Supplementary Table 3. List of antibodies and dyes.

When the concentration of the stock antibody or the dye solution were not available, the dilution is declared.

Antibody or dye	Manufacturer	Reference	Western or dot blot concentration (µg / µl)	IF concentration (µg / µl)

Anti Munc18-2	Abcam	ab103976	1	NA
Anti Sodium Potassium ATPase α1	Abcam	ab283318	NA	5
Anti Tspan-8	Abcam	ab70007	1	10
Anti VAMP-8	Abcam	ab76021	0.2	NA
Anti TGN46	abd serotec	AHP500	NA	1
CellBrite Red	Biotium	BT-30023	NA	1:200
Anti Rab11A	Cell Signaling	2413	0.5	NA
Anti syntaxin-1 (HT29-N2)	Chemicon	AB5820	1:1000	NA
Anti syntaxin-3 (HT29-N2)	Chemicon	AB5448	1:1000	NA
Anti syntaxin 4 (HT29-N2)	Chemicon	AB5330	1:1000	NA
Anti RFP	Evrogen	AB233	20	NA
Anti RFP (clone 5F8) (Used for INS-1 exp)	Chromotek now Protein Tech	5f8-20	1	NA
Anti mucin5-AC (clone 45M1)	Labvision Neomarkers now part of ThermoFisher Scientific	MS-145-P0	0.04	0.2

DAPI	Invitrogen	D3571	NA	0.5
Anti syntaxin-2 (HT29-N2)	ProteinTech	55033-1-AP	NA	10
Anti GFP	Roche	11814460001	0.4	0.8
Anti GRASP65	Santa Cruz	sc-19481	NA	1
Anti Lamp-1	Santa Cruz	sc-18821	NA	0.02
Anti beta-tubulin	Sigma-Aldrich	T4026	1:5000	NA
Anti Munc18-1	Synaptic Systems	116 003	1	NA
Anti syntaxin-1A (INS-1)	Synaptic Systems	110 118	1	NA
Anti syntaxin-2 (HT29-N2)	Synaptic Systems	110 123	1	NA
Donkey anti rabbit IgG - Alexa Fluor 680	Life technologies	A10043	NA	1 µg/mL
Donkey anti mouse IgG – Alexa Fluor Plus 800	Invitrogen	A32789	NA	1 µg/mL

Supplementary figures

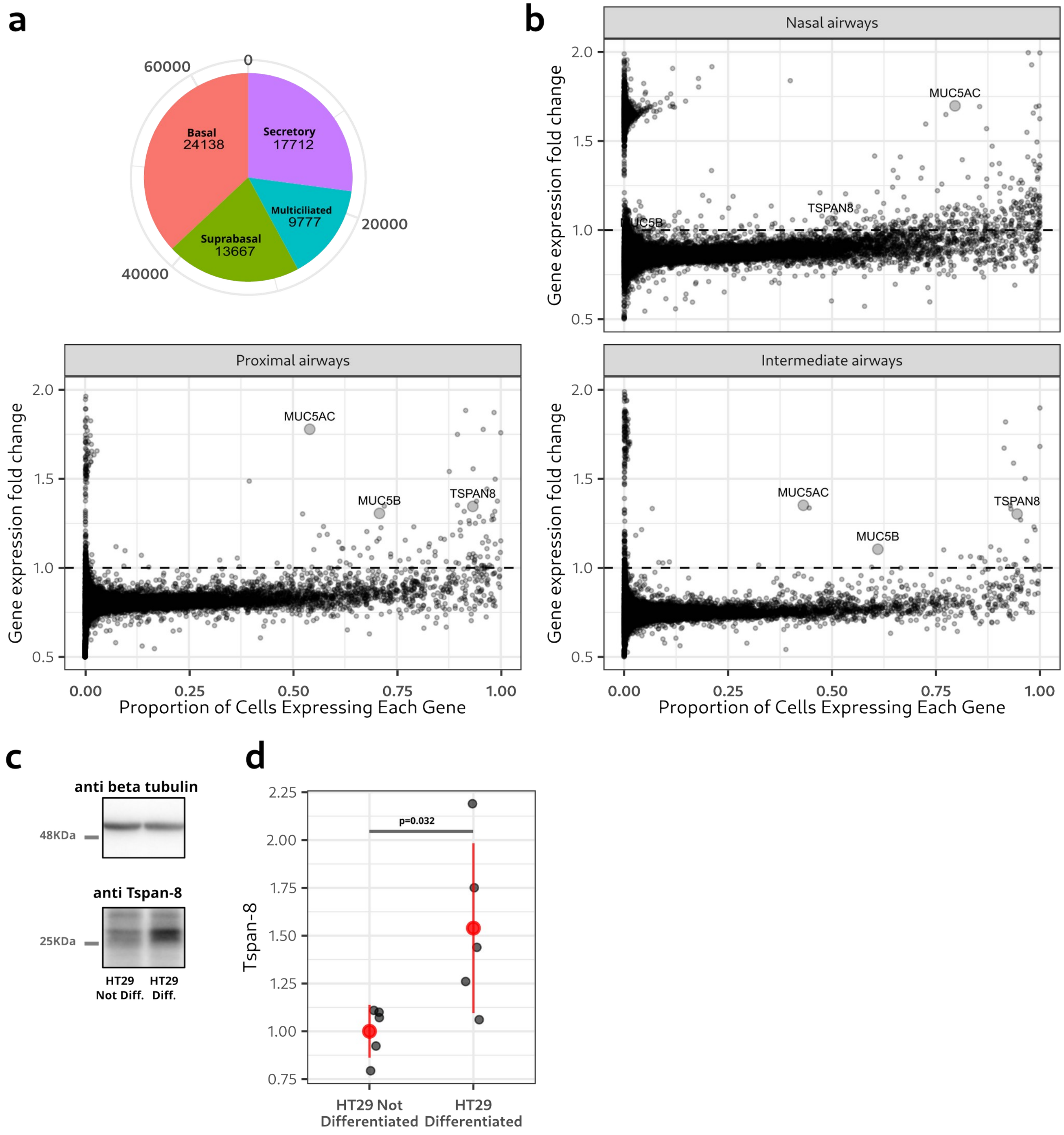

Supplementary Fig. 1. Tetraspanin-8 enrichment in mucin-secreting cells from the healthy human airways.

a. Pie chart showing the total number and type of cells analyzed in the bioinformatics analysis.

b. Dot plots showing the fold-change in gene expression between undifferentiated basal and mucin-secreting cells in the y-axis and the proportion of mucin-secreting cells expressing each gene in the x-axis. Each dot represents one gene. Larger dots represent MUC5AC, MUC5B and TSPAN8. Genes below the dashed horizontal line are down regulated in mucin secreting cells while genes above the line are up

regulated. Dots were plotted with a 50% transparency value for better visibility in areas of the graph with high dots density.

c. Representative western blot of the total amount of beta-tubulin (upper panel) and Tspan-8 (lower panel) detected in growing (non-differentiated) and differentiated HT29-N2 (mucin-secreting) cells. Blots were incubated with anti beta-tubulin and anti Tspan-8 antibodies and developed by enhanced chemiluminescence (ECL).

d. Quantification of the total amount of beta-tubulin and Tspan-8 in growing (non-differentiated) and differentiated mucin secreting HT29-N2 cells. A representative membrane is shown in **C.** Each dot represents the signal of an independent replicate. Total number of replicates are 5. Red dots represent the mean +/- the standard deviation. The p value is from a one-way ANOVA analysis.

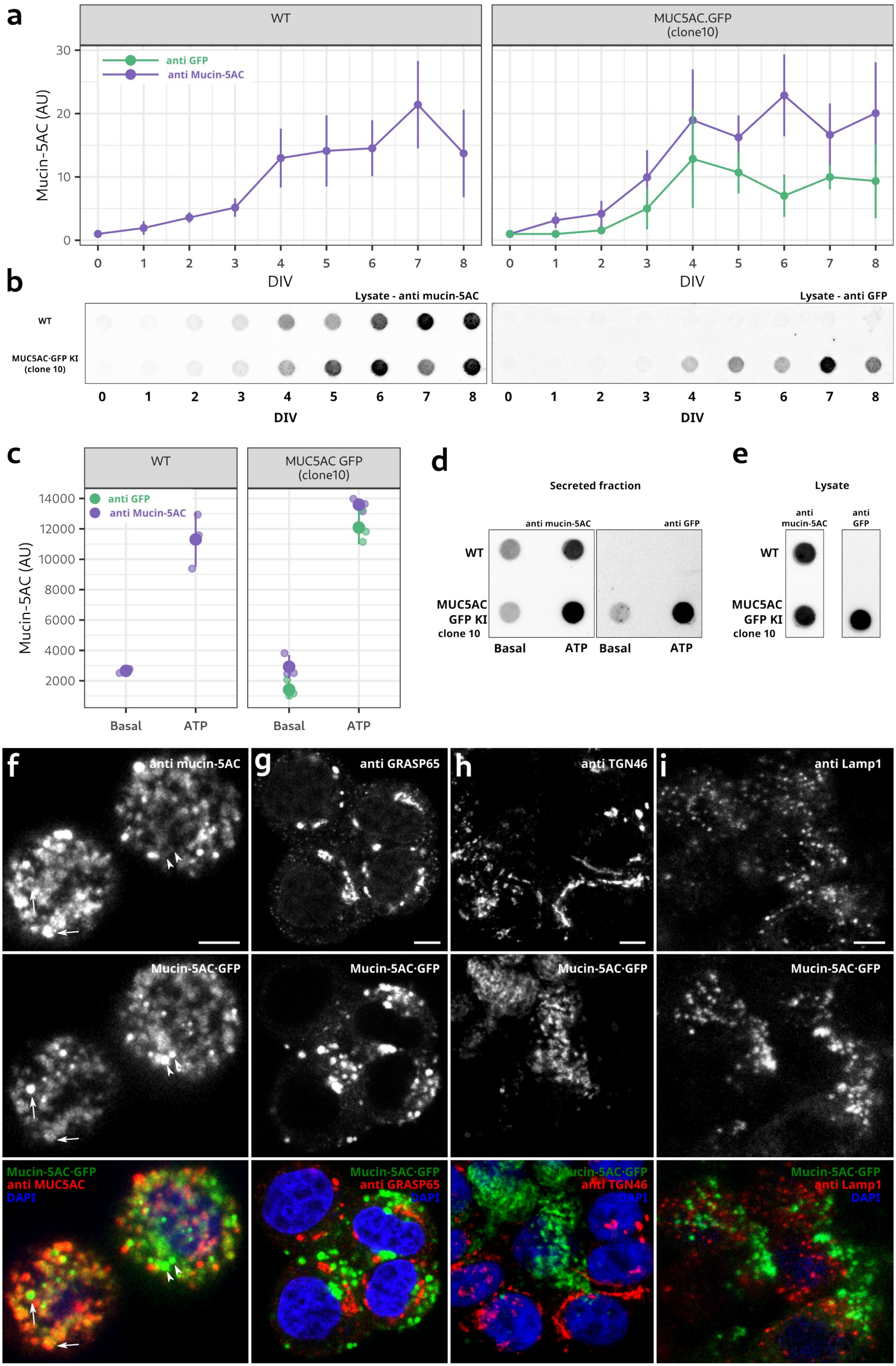
a
WT
MUC5AC.GFP
(clone10)
anti GFP
anti Mucin-5AC
Mucin-5AC (AU)
DIV
b
Lysate - anti mucin-5AC
Lysate - anti GFP
WT
MUC5AC·GFP KI
(clone 10)
DIV
c
WT
MUC5AC GFP
(clone10)
anti GFP
anti Mucin-5AC
Mucin-5AC (AU)
Basal
ATP
d
Secreted fraction
anti mucin-5AC
anti GFP
WT
MUC5AC
GFP KI
clone 10
Basal
ATP
Basal
ATP
e
Lysate
anti
mucin-5AC
anti
GFP
WT
MUC5AC
GFP KI
clone 10
f
anti mucin-5AC
g
anti GRASP65
h
anti TGN46
i
anti Lamp1
Mucin-5AC·GFP
Mucin-5AC·GFP
anti MUC5AC
DAPI
anti GRASP65
anti TGN46
anti Lamp1

Supplementary Fig. 2. Characterization of the MUC5AC CRISPR/Cas9 sfGFP KI cell line.

a. Quantification of the total amount of mucin-5AC during HT29-N2 cell line differentiation as detected by immunolabeling of mucin-5AC (purple line and dots) and GFP emission (green lines and dots). Representative dot blots membranes are shown in **b**. Each dot represents the mean value of three independent experiments +/- the standard deviation. For each replicate, mucin-5AC values of cells in culture for 1 to 8 days *in vitro* (DIV) were relativized to the value of non-differentiated cells (0 DIV).

b. Representative dot blots showing the total amount (cell lysates) of mucin-5AC in HT29-N2 cells during differentiation. The left western blot membrane was incubated with an anti mucin-5AC antibody. The right membrane was loaded with identical samples to the left membrane and was incubated with an anti GFP antibody.

c. Quantification of the amount of secreted mucin-5AC by WT and MUC5AC sfGFP KI (clone 10) cells. A representative dot blot is shown in **d**. Purple dots represent the signal of anti mucin-5AC immunoblotting. Green dots represent the signal from anti GFP immunoblotting. Smaller dots represent the mucin-5AC amount detected from 3 independent secretion assays. All samples were processed in parallel. Bigger dots represent the mean secreted mucin-5AC +/- the standard deviation. A two-factor ANOVA analysis showed no statistical difference between WT and mucin-5AC·GFP cell lines ($p = 0.696$).

d. Representative dot blots of a mucin secretion assay of WT and MUC5AC sfGFP KI (clone 10) cells.

e. Representative dot blots showing the total amount (cell lysate) of mucin-5AC in differentiated WT and mucin-5AC·GFP-expressing (clone 10) cells. The left membrane was immunoblotted with an anti mucin-5AC antibody. The right membrane was loaded with identical samples as in the left membrane and was immunoblotted with an anti GFP antibody.

f – i. Top row: Optical planes from confocal images of differentiated mucin-5AC·GFP (clone 10) cells and immunolabeled for mucin-5AC **(f)**, GRASP65 **(g)**, TGN46 **(h)** and Lamp1 **(i)**. Scale bar is 10 µm. Arrows in **f** point to co-localizing anti mucin-5AC and mucin-5AC·GFP signals. Arrowheads point to mucin-5AC·GFP-positive but anti mucin-5AC-negative granules. In the merged images DAPI was used to visualize the cell nucleus. Five images from two independent cell cultures were taken. One is shown. All images showed similar results.

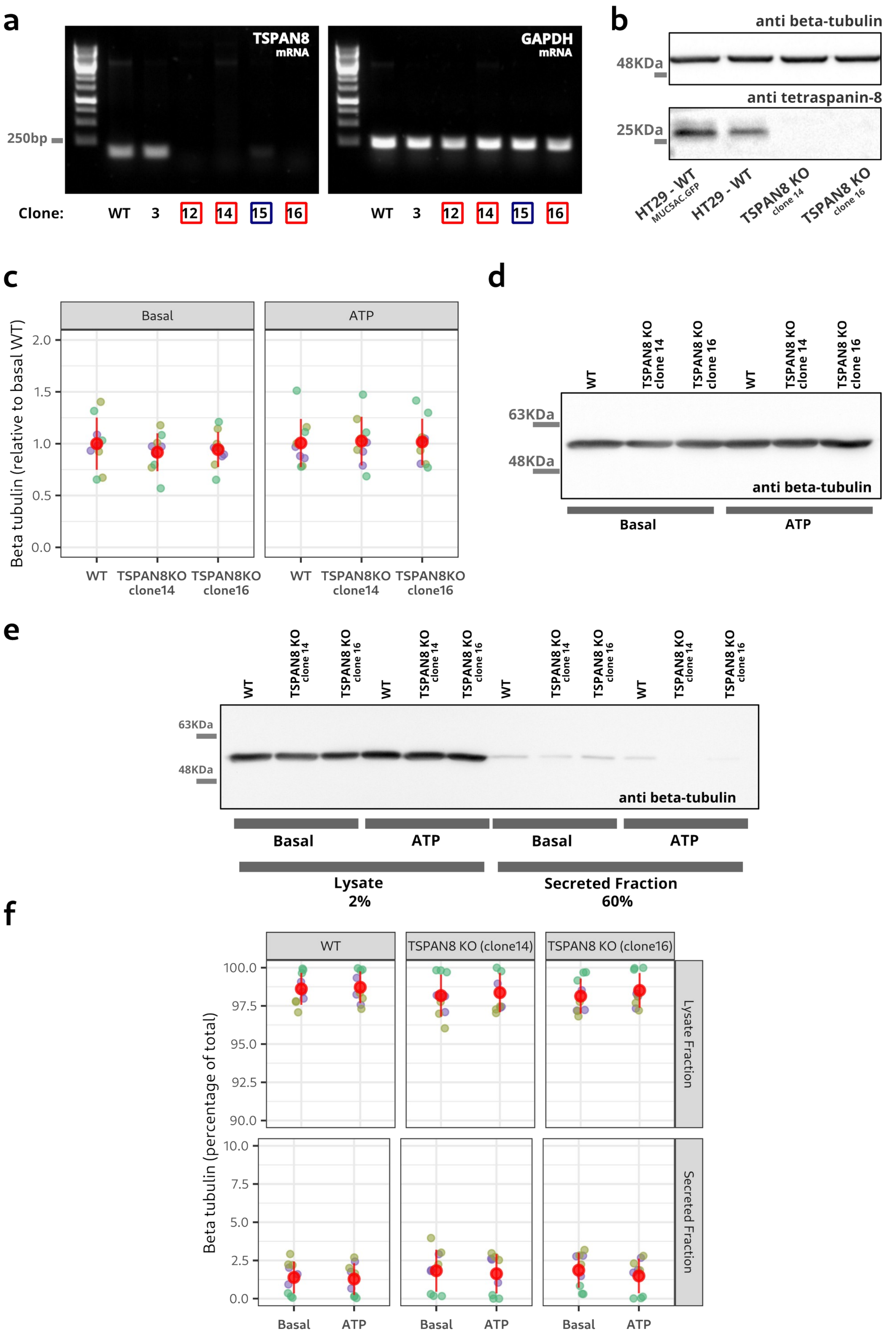
a
TSPAN8 mRNA
GAPDH mRNA
250bp
Clone: WT 3 12 14 15 16
WT 3 12 14 15 16
b
anti beta-tubulin
48KDa
anti tetraspanin-8
25KDa
HT29 - WT MUC5AC.GFP
HT29 - WT
TSPAN8 KO clone 14
TSPAN8 KO clone 16
c
Basal
ATP
Beta tubulin (relative to basal WT)
2.0
1.5
1.0
0.5
0.0
WT
TSPAN8KO clone14
TSPAN8KO clone16
d
WT
TSPAN8 KO clone 14
TSPAN8 KO clone 16
63KDa
48KDa
anti beta-tubulin
Basal
ATP
e
63KDa
48KDa
anti beta-tubulin
Basal
ATP
Lysate 2%
Secreted Fraction 60%
f
WT
TSPAN8 KO (clone14)
TSPAN8 KO (clone16)
Beta tubulin (percentage of total)
100.0
97.5
95.0
92.5
90.0
10.0
7.5
5.0
2.5
0.0
Lysate Fraction
Secreted Fraction
Basal
ATP

Supplementary Fig. 3. Tetraspanin 8 KO generation and secretion assays.

a. CRISPR/Cas9-genetically modified cell lines were screened for the presence of TSPAN8 mRNA. Agarose gels showing TSPAN8 and GAPDH cDNA amplification. cDNA was obtained by RT-PCR from purified mRNA. Red boxes show full TSPAN8 KO and the purple box shows a probable heterozygote cell line. GAPDH was used as a positive RT-PCR control.

b. Representative western blot showing the total amount of Tspan-8 in WT and TSPAN8 KO (clones 14 and 16) cell lines. Beta-tubulin was used as loading control.

c. Quantification of the total (cell lysates) beta-tubulin content of the secretion assays quantified in Fig. **2b**. A representative western blot is shown in **d**. Each dot represents beta-tubulin signal from independent secretion assays. Grouped in different colors are secretion assays that were processed in parallel. Total number of replicates are 9. Red dots represent the mean +/- the standard deviation. Values are expressed as relative to the average beta-tubulin content of non-stimulated WT cells. A two-way ANOVA with interaction was done, and no statistical differences were found.

d. Loading control of a dot blot shown in Fig. **2a** and quantified in Fig. **2b**.

e. Representative western blot of the amount of beta-tubulin present in the cell lysates and in the secreted fractions of a secretion assay quantified in Fig. **2b**. 2% of the cell lysate and 60% of the secreted fraction were loaded into the gel. The secreted fraction was precipitated with trichloro acetic acid to reduce the total volume of the sample.

f. Quantification of the amount of beta-tubulin present in cell lysates and in the secreted fractions of a secretion assay quantified in Fig. **2b**. A representative western blot is shown in **e**. Each dot represents the beta-tubulin signal from independent secretion assays. Grouped in different colors are secretion assays that were processed in parallel. Total number of replicates are 9. Red dots represent the mean +/- the standard deviation. Values are expressed as percentage of the total (lysate and secreted) amount of beta-tubulin. A two-way ANOVA with interaction was done, and no statistical differences were found.

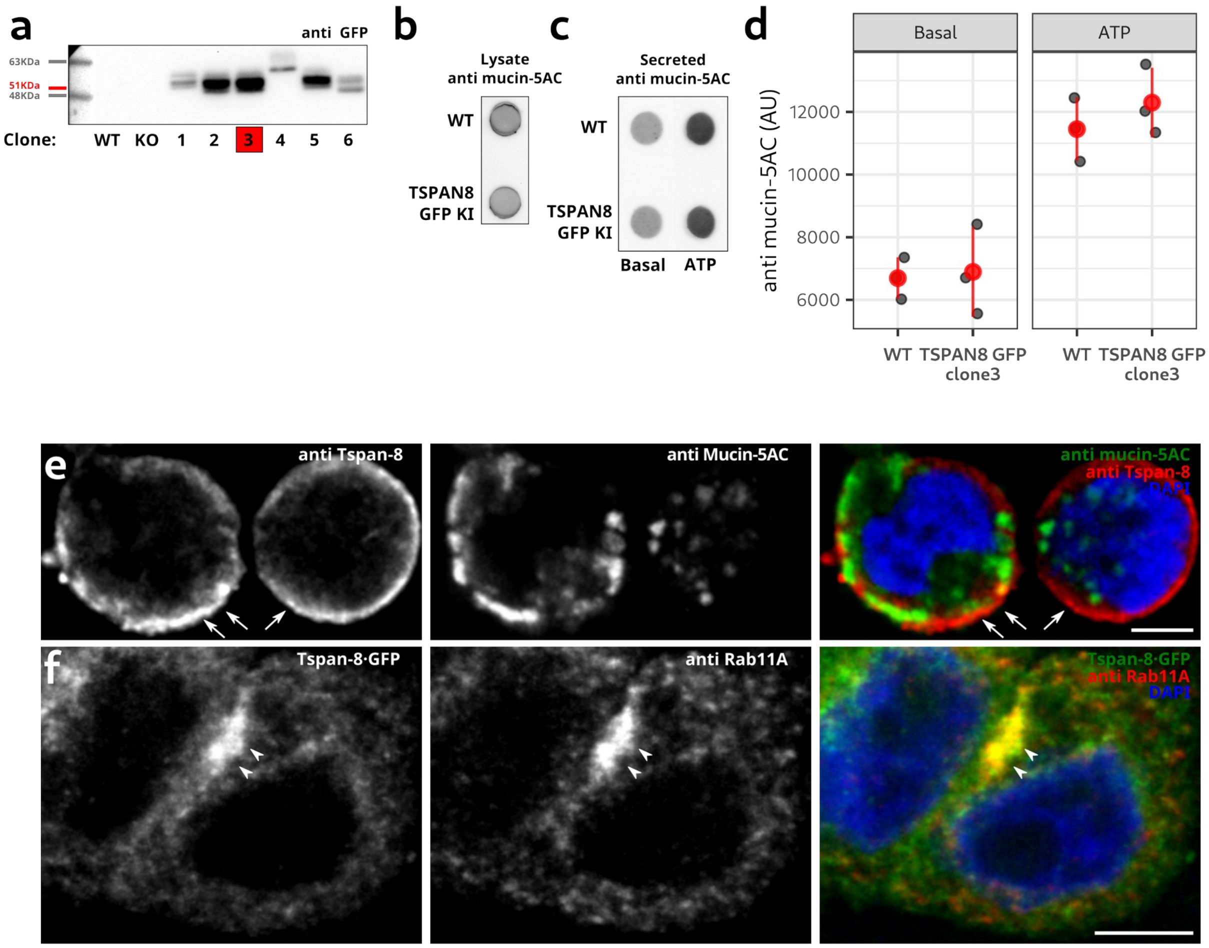

Supplementary Fig. 4. Generation of a tetraspanin 8·GFP-expressing cell line by CRISPr/Cas.

a. Western blot screening of 6 different cell lines modified to express sfGFP at the c-terminus of TSPAN8. Lysates from WT and TSPAN8 KO cells were used as control. The red tick shows the expected migration pattern of the fusion protein Tspan8·GFP (26KDa from Tspan-8 and 25KDa from sfGFP). Clone 3 was used for all subsequent experiments.

b. Representative dot blot showing the mucin-5AC content of WT and Tspan-8·GFP (clone 3) cell lysates. The membrane was immunoblotted with an anti mucin-5AC antibody.

c. Representative dot blot of a secretion assay showing mucin-5AC content in the secreted fractions of WT and Tspan-8·GFP (clone 3) cell lines. The membrane was immunoblotted with an anti mucin-5AC antibody. The signal was detected by fluorescence emission.

d. Quantification of the secretion assays of WT and Tspan-8·GFP (clone 3) cell lines. A representative dot blot is shown in **c**. Each gray dot represents the mucin-5AC amount of an independent secretion assay. All three secretion assays were run in parallel. The red dot is the mean value of the gray dots +/- the standard deviation. The y-axis is in arbitrary units (AU). A two-way ANOVA with interaction of the secreted mucin-AC was done and no statistical differences were found.

e. Optical plane from a confocal image of WT mucin-secreting cells immunolabelled for Tspan-8 and mucin-5AC. DAPI was used to visualize the cell nucleus in the merge of the two. Arrows point to a region of the cell that is not proximal to the cell nucleus. Scale bar is 10 µm. Five images from two independent cell cultures were taken. One is shown. All images showed similar results.

f. Optical image of a representative confocal image of a mucin-secreting cell expressing Tspan-8·GFP at endogenous levels and immunolabelled against Rab11A. DAPI was used to visualize the cell nucleus in the merge of the two. The GFP signal was enhanced by antibody detection of GFP. Arrowheads point to

recycling endosomes as determined by its localization and Rab11A signal. Scale bar is 10 μm. Seven images from two independent cell cultures were taken. One is shown. All images showed similar results.

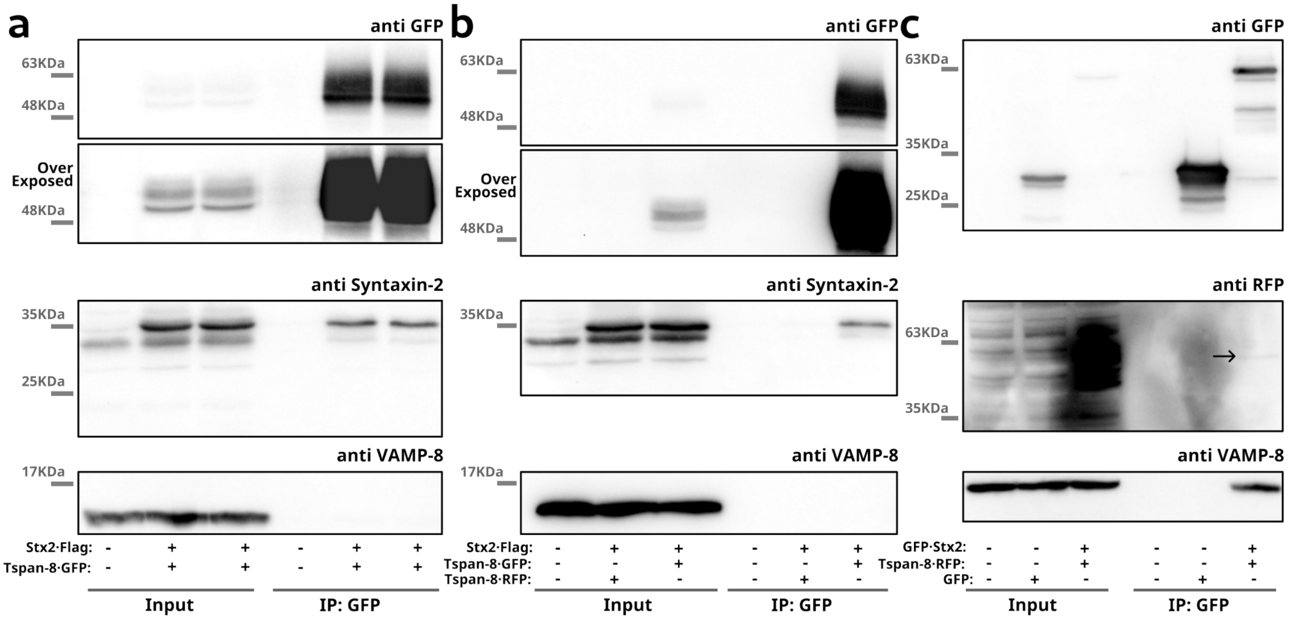

Supplementary Fig. 5. Tspan-8 interacts with syntaxin-2.

a-b. Lysates of WT HT29-N2 cells transiently co-transfected with Tspan-8·GFP and Stx2·FLAG were processed for GFP immunoprecipitation and western blot analysis. Top panels show immunoblotting against GFP to confirm the immunoprecipitation. No transfection (**a** and **b**) and Tspan-8·RFP transfection (**b**) were used as control conditions. In **a**, 2 independent test conditions are shown in the same membrane.

c. Lysates of WT HT29-N2 cells transiently co-transfected with GFP·Stx2 and Tspan-8·RFP were processed for GFP immunoprecipitation and western blot analysis. Top panel shows immunoblotting against GFP to confirm the immunoprecipitation. No transfection and transfection of soluble GFP were used as control conditions. The arrow points to the Tspan-8·RFP band on the co-immuno-precipitate. One of 2 independent experiment is shown. Both replicates showed similar results.

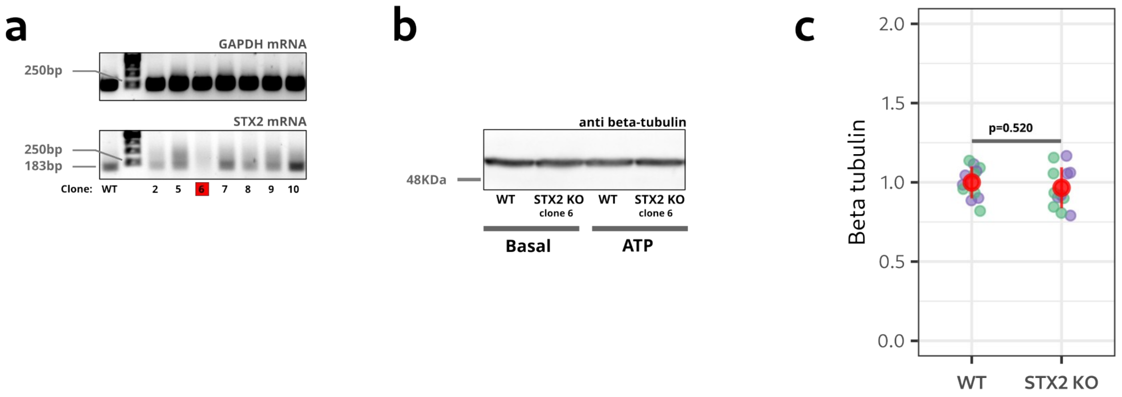

Supplementary Fig. 6. Syntaxin-2 in necessary for mucin-5AC secretion.

a. Grown cell lines identified as clones 2, 5, 6, 7, 8, 9 and 10 were screened for the presence of STX2 mRNA after CRISPR/Cas9 genome editing. cDNA was obtained by RT-PCR from the mRNA purified of the screened cell lines. Red box shows a full STX2 KO clone. GAPDH was used as a positive RT-PCR control. Clone 6 was used for subsequent experiments.

b. Representative western blot of the total amount (cell lysate) of beta-tubulin of the secretion assay shown in Fig. **5i**. The membrane was immunoblotted with an anti beta-tubulin antibody and developed by ECL.

c. Quantification of the total (cell lysates) beta-tubulin content from the samples of the secretion assays quantified in Fig. **5j**. A representative western blot is shown in **b**. Each dot represents beta-tubulin signal from a single secretion assay. Grouped in different colors are samples from basal and ATP-stimulated cells. Total number of replicates are 9. Red dots represent the mean +/- the standard deviation. Values are expressed as relative to the average beta-tubulin content in WT cells. A two-way ANOVA with interaction was done, and no statistical differences were found. Genotype:Secretion interaction p value = 0.757; Secretion principal factor p value = 0.742; Genotype principal factor p value = 0.520.

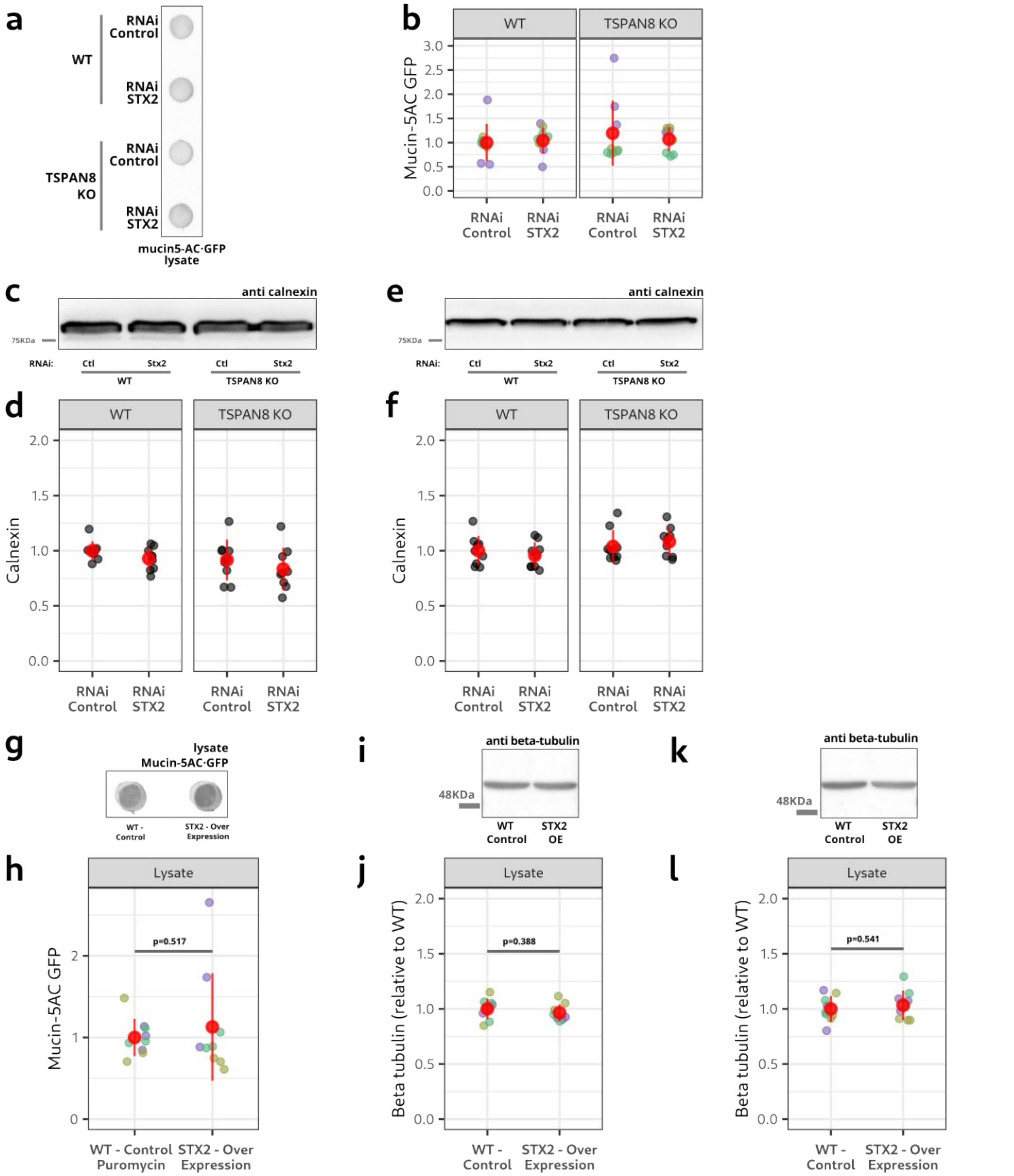

Supplementary Fig. 7. Syntaxin-2 and Tspan-8 are in the same pathway of regulated mucin secretion.

a. Representative dot blot of the total (cell lysate) mucin-5AC·GFP in WT and TSPAN8 KO cells treated with RNAi control and RNAi against STX2. The fluorescent emission of sfGFP is shown.

b. Quantification of the amount of mucin-5AC·GFP in the cell lysates of WT and TSPAN8 KO cells treated with RNAi control and RNAi against STX2. A representative dot blot is shown in **a**. Each dot represents signal from an independent sample. Grouped in different colors are samples that were processed in parallel. Total number of replicates are 9. Red dots represent the mean +/- the standard deviation. A two-way ANOVA with interaction was done, and no statistical differences were found.

c. Loading control of the dot blot shown in **a**. Representative western blot of the total amount (cell lysate) of calnexin.

d. Quantification of the total (cell lysates) calnexin content from the samples used in the dot blots quantified in **b**. A representative western blot is shown in **c**. Each dot represents calnexin signal from an independent sample. Total number of replicates are 9. Red dots represent the mean +/- the standard deviation. Values are expressed as relative to the average calnexin content in WT control cells. A two-way ANOVA with interaction was done, and no statistical differences were found.

e. Loading control of the secretion assay shown in Fig. **6f**. Representative western blot of the total amount (cell lysate) of calnexin.

f. Quantification of the total (cell lysates) calnexin content of the secretion assays quantified in Fig. **6g**. A representative western blot is shown in **e**. Each dot represents calnexin signal from a single secretion assay. Total number of replicates are 9. Red dots represent the mean +/- the standard deviation. Values are expressed as proportions relative to the average calnexin content in WT control cells. A two-way ANOVA with interaction was done, and no statistical differences were found.

g. Representative dot blot of the total (cell lysate) mucin-5AC·GFP detected in control and STX2-over expressing cells.

h. Quantification of the detected amount of mucin-5AC·GFP in the cell lysates of control and STX2-over expressing cells. A representative dot blot is shown in **g**. Each dot represent the signal from a single sample. Grouped in different colors are samples that were processed in parallel. Total number of replicates are 9. Red dots represent the mean +/- the standard deviation. A one-way ANOVA was done and the p value is shown in the graph.

i. Loading control of the dot blot shown in **g**. Representative western blot of the total amount (cell lysate) of beta-tubulin. OE refers to over expression.

j. Quantification of the total (cell lysates) beta-tubulin content from the samples used in the dot blots quantified in **h**. A representative western blot is shown in **i**. Each dot represents the beta-tubulin signal from a single sample. Grouped in different colors are samples that were processed in parallel. Total number of replicates are 9. Red dots represent the mean +/- the standard deviation. Values are expressed as relative to the average beta-tubulin content in WT control cells. A one-way ANOVA was done and the p value is shown in the graph.

k. Loading control of the dot blot shown in Fig. **6j**. Representative western blot of the total amount (cell lysate) of beta-tubulin.

l. Quantification of the total (cell lysates) beta-tubulin content from the secretion assay quantified in Fig. **6k**. A representative western blot is shown in **i**. Each dot represents the beta-tubulin signal from an independent secretion assay. Grouped in different colors are secretion assays that were run in parallel. Total number of replicates are 9. Red dots represent the mean +/- the standard deviation. Values are expressed as relative to the average beta-tubulin content in WT control cells. A one-way ANOVA was done and the p value is shown in the graph.
