# Supplementary material for: Tetraspanin-8 sequesters syntaxin-2 to control biphasic release propensity of mucin granules
Source: Nat Commun. 2023 Jun 22;14:3710. doi: 10.1038/s41467-023-39277-9 (PMC10287693; doi:10.1038/s41467-023-39277-9)
Supplement: Supplementary file 3 — Reporting Summary [file 41467_2023_39277_MOESM3_ESM.pdf]

nature portfolio

Corresponding author(s): Malhotra Vivek

Last updated by author(s): May 21, 2023

Reporting Summary

Nature Portfolio wishes to improve the reproducibility of the work that we publish. This form provides structure for consistency and transparency in reporting. For further information on Nature Portfolio policies, see our Editorial Policies and the Editorial Policy Checklist.

Statistics

For all statistical analyses, confirm that the following items are present in the figure legend, table legend, main text, or Methods section.

n/a	Confirmed	
☐	☒	The exact sample size (*n*) for each experimental group/condition, given as a discrete number and unit of measurement
☐	☒	A statement on whether measurements were taken from distinct samples or whether the same sample was measured repeatedly
☐	☒	The statistical test(s) used AND whether they are one- or two-sided<br>*Only common tests should be described solely by name; describe more complex techniques in the Methods section.*
☒	☐	A description of all covariates tested
☐	☒	A description of any assumptions or corrections, such as tests of normality and adjustment for multiple comparisons
☐	☒	A full description of the statistical parameters including central tendency (e.g. means) or other basic estimates (e.g. regression coefficient) AND variation (e.g. standard deviation) or associated estimates of uncertainty (e.g. confidence intervals)
☐	☒	For null hypothesis testing, the test statistic (e.g. *F*, *t*, *r*) with confidence intervals, effect sizes, degrees of freedom and *P* value noted<br>*Give P values as exact values whenever suitable.*
☒	☐	For Bayesian analysis, information on the choice of priors and Markov chain Monte Carlo settings
☒	☐	For hierarchical and complex designs, identification of the appropriate level for tests and full reporting of outcomes
☐	☒	Estimates of effect sizes (e.g. Cohen's *d*, Pearson's *r*), indicating how they were calculated

Our web collection on statistics for biologists contains articles on many of the points above.

Software and code

Policy information about availability of computer code

Data collection	No software was used to collect data.
Data analysis	The software R (v4.2) was used to compute all statistical tests and plots.<br>The R code to reproduce the bioinformatics analysis of the data published by Deprez et. al. 2020 is publicly available on GitHub under the GNU General Public license version 3 [https://doi.org/10.5281/zenodo.7954818].<br>Icy software (v2.4) and Fiji/Imagej (v1.5) were used for image analysis.

For manuscripts utilizing custom algorithms or software that are central to the research but not yet described in published literature, software must be made available to editors and reviewers. We strongly encourage code deposition in a community repository (e.g. GitHub). See the Nature Portfolio guidelines for submitting code & software for further information.

Data

Policy information about availability of data

All manuscripts must include a data availability statement. This statement should provide the following information, where applicable:

- Accession codes, unique identifiers, or web links for publicly available datasets
- A description of any restrictions on data availability
- For clinical datasets or third party data, please ensure that the statement adheres to our policy

Source data are provided with this paper. In the source data file, raw quantification data and annotated raw images of the blots shown in the manuscript are

provided in a spreadsheet. The raw data of each figure is placed in a tab with the corresponding figure name.
The raw data generated in this study has been deposited in a Zenodo repository under accession code 7680682 and is publicly available under Creative Commons Attribution 4.0 International license [https://doi.org/10.5281/zenodo.7680682].
The data for the bioinformatics analysis has been previously reported by Deprez et.at. 2020 and is publicly available through an interactive web tool [https://www.genomique.eu/cellbrowser/HCA/].

Human research participants

Policy information about studies involving human research participants and Sex and Gender in Research.

Reporting on sex and gender	NA
Population characteristics	NA
Recruitment	NA
Ethics oversight	NA

Note that full information on the approval of the study protocol must also be provided in the manuscript.

Field-specific reporting

Please select the one below that is the best fit for your research. If you are not sure, read the appropriate sections before making your selection.

☒ Life sciences ☐ Behavioural & social sciences ☐ Ecological, evolutionary & environmental sciences

For a reference copy of the document with all sections, see nature.com/documents/nr-reporting-summary-flat.pdf

Life sciences study design

All studies must disclose on these points even when the disclosure is negative.

Sample size	Sample size were not pre-calculated. Sample size were pre-determined between 9 and 15 as this gives us the statistical power to detect significant differences if there were.
Data exclusions	No data was excluded from the statistical tests.
Replication	Replicates were done so in every experiment there was at least 9 statistical independent samples. If samples were run in parallel they were nested together in the statistical model to control for potential variation associated with the particular batch of samples.<br>All replications attemps gave similar results and are included in the statistical analysis and graphical representation of the data.
Randomization	Cell samples were randomly allocated to the different treatments.
Blinding	For all microscopic analysis, the researchers were blind to the treatment associated to the samples. Quantification was done by scripting in Icy and ImageJ therefore unbiased. Researchers were blinded for experimental group allocation in Western Blot analysis, ELISA determination of insulin content in the culture media and any other experimental analysis conducted in this work.

Reporting for specific materials, systems and methods

We require information from authors about some types of materials, experimental systems and methods used in many studies. Here, indicate whether each material, system or method listed is relevant to your study. If you are not sure if a list item applies to your research, read the appropriate section before selecting a response.

Materials & experimental systems

n/a	Involved in the study	
☐	☒	Antibodies
☐	☒	Eukaryotic cell lines
☒	☐	Palaeontology and archaeology
☒	☐	Animals and other organisms
☒	☐	Clinical data
☒	☐	Dual use research of concern

Methods

n/a	Involved in the study	
☒	☐	ChIP-seq
☒	☐	Flow cytometry
☒	☐	MRI-based neuroimaging

Antibodies

Antibodies used

Anti Munc18-2 Abcam ab103976
Anti Sodium Potassium ATPase α1 Abcam ab283318
Anti Tspan-8 Abcam ab70007
Anti VAMP-8 Abcam ab76021
Anti TGN46 abd serotec AHP500
CellBrite Red Biotium BT-30023
Anti Rab11A Cell Signaling 2413
Anti syntaxin-1 Chemicon AB5820
Anti syntaxin-3 Chemicon AB5448
Anti syntaxin 4 Chemicon AB5330
Anti RFP Evrogen AB233
Anti RFP (clone 5F8) Chromotek now Protein Tech 5f8-20
Anti mucin5-AC (clone 45M1) Labvision Neomarkers now part of ThermoFisher Scientific MS-145-P0
DAPI Invitrogen D3571
Anti syntaxin-2 ProteinTech 55033-1-AP
Anti GFP Roche 11814460001
Anti GRASP65 Santa Cruz sc-19481
Anti Lamp-1 Santa Cruz sc-18821
Anti beta-tubulin Sigma-Aldrich T4026
Anti Munc18-1 Synaptic Systems 116 003
Anti syntaxin-1A Synaptic Systems 110 118
Anti syntaxin-2 Synaptic Systems 110 123
Donkey anti rabbit IgG – Alexa Fluor 680 Life technologies A10043
donkey anti mouse IgG – Alexa Fluor Plus 800 Invitrogen A32789

Validation

Anti Munc18-2 was validated by the manufacturer: Western blot of H1299 and HeLa whole cell lysate.
Anti Sodium Potassium ATPase α1 was validated by the manufacturer: Western blot of RAW264.7, C6 (rat glial tumor glial cell), and HeLa whole cell lysate and Immunofluorescence.
Anti Tspan-8 was validated by the Malhotra laboratory with a TSPAN8 HT29-N2 KO cell line.
Anti VAMP-8 was validated by the manufacturer: Western blot of WT and VAMP8 KO HEK-293T whole cell lysate.
Anti TGN46 was validated in the Malhotra laboratory by immuno staining several cell lines (HT29-N2, HEK-293T, U2OS) and localizing the signal to the Trans Golgi Network.
CellBrite Red was validated by the Malhotra laboratory by labelling cells in culture (HT29-N2 and HEK-293T) and localizing the signal to the plasma membrane.
Anti Rab11A was validated by the manufacturer: Western blot of PC-12, COS, C2C12 and A549 whole cell lysate and immunofluorescence of HeLa cells.
Anti syntaxin-1 was validated by the manufacturer: Western blot of rat brain membranes.
Anti syntaxin-3 was validated by the manufacturer: Western blot of rat brain membranes.
Anti syntaxin-4 was validated by the manufacturer: Western blot of rat brain membranes.
Anti RFP (Evrogen) was validated in the Malhotra laboratory by Western Blot and immunofluorescence of cells transfected with RFP.
Anti RFP (clone 5F8) was validated in the Malhotra laboratory by Western Blot and immunofluorescence of cells transfected with RFP.
Anti mucin5-AC antibody was validated by the Malhotra laboratory by IF of mucin5-AC.GFP tagged HT29-N2 cells.
DAPI was validated in the Malhotra laboratory by labelling HT29-N2, U2OS and HEK-293T cells and microscopic analysis.
Anti syntaxin-2 was validated by the manufacturer: Western blot of A432 and HeLa whole cell lysate and Immunofluorescence of HeLa cells.
Anti GFP was validated by the Malhotra laboratory by Western Blot and immunofluorescence of cells transfected with GFP.
Anti GRASP65 was validated in the Malhotra laboratory by immuno staining several cell lines (HT29-N2, HEK-293T, U2OS) and localizing the signal to the Golgi apparatus.
Anti Lamp-1 was validated by the manufacturer: Western blot analysis of LAMP-1 expression in HeLa, JAR, ECV304, U-937, Jurkat and Ramos whole cell lysates and HeLa immunofluorescence.
Anti beta-tubulin validated in the Malhotra laboratory by Western Blot of HT29-N2, HEK-293T and U2OS hole cell lysates and immunofluorescence of HT29-N2.
Anti Munc18-1 was validated by the manufacturer: Western blot analysis of crude synaptosomal fraction of rat brain and immunofluorescence of primary neurons in culture.
Anti syntaxin-1A was validated by the manufacturer: Western blot analysis of rat brain lysates and immunofluorescence of primary neurons in culture.
Anti syntaxin-2 was validated by the manufacturer: Western blot analysis of rat brain homogenate and immunofluorescence of HeLa cells in culture.
Donkey anti rabbit IgG – Alexa Fluor 680 was validated by the manufacturer: Western blots of nuclear extracts of HeLa and U-87 MG. The blots were probed with Anti-JUNB Recombinant Rabbit Monoclonal Antibody (Product # 701702, 2 µg/mL) and detected using Donkey Anti-Rabbit IgG (H+L) Secondary Antibody, Alexa Fluor 680. Immunofluorescence of Donkey anti-Rabbit IgG (H+L) Secondary Antibody, Alexa Fluor 680 was performed using A-431 cells stained with EGFR (EP38Y) Rabbit Monoclonal Primary Antibody (Product # MA5-14485).
donkey anti mouse IgG – Alexa Fluor Plus 800 was validated by the manufacturer: Western blots of whole cell extracts of MCF 10A, SH-SY5Y, HaCaT, HeLa and MCF7 cell lines. Immunofluorescence analysis of HEK 293 cells using primary antibodies against Pericentrin (Product # PA5-53498), DDX5 (Product # PA1-31019) and ATP5A1 (Product # 43-9800).

Eukaryotic cell lines

Policy information about cell lines and Sex and Gender in Research

Cell line source(s)	HT29-N2 cells (obtained from ATCC) (RRID: CVCL_5942)<br>Caco2 (ATCC - HTB-37)<br>Rat insulinoma cell line (INS-1. Sigma-aldrich - SCC208)<br>HEK-293T (ATCC)
Authentication	HT29-N2 cell line was verified by mucin production and secretion.<br>INS-1 cells were verified by insulin production and secretion upon glucose stimulation.<br>HEK-293T and Caco2 cell lines were not verified.
Mycoplasma contamination	In the Center of Genomic Regulation a compulsory mycoplasma test has to be done every 2 months. All our cell lines included in our work were negative for the presence of mycoplasma contamination.
Commonly misidentified lines<br>(See ICLAC register)	No commonly misidentified lines were used.
