# Supplementary material for: Tetraspanin-8 sequesters syntaxin-2 to control biphasic release propensity of mucin granules
Source: Nat Commun. 2023 Jun 22;14:3710. doi: 10.1038/s41467-023-39277-9 (PMC10287693; doi:10.1038/s41467-023-39277-9)
Supplement: Supplementary file 4 — Description of additional supplementary files [file 41467_2023_39277_MOESM4_ESM.pdf]

Description of additional supplementary files

Supplementary Video 1 : GFP-tagged mucin5-AC is secreted after ATP stimulation. Spinning disk confocal imaging of CRISP/Cas genetically-modified HT29-N2 cells to express mucin5- AC·GFP under endogenous regulation. Cells were imaged during 5 minutes without stimulation. ATP addition is marked by red ATP in the top left region of the time-lapse at time 0. Each white dot in the time-lapse is a mucin granule. Each image was taken every 20 seconds. Shown is the maximal projection of the z-stack.

Supplementary Video 2 : Time-lapse of WT and TSPAN8 KO HT29-N2 cells during ATP stimulation. WT and TSPAN8 KO HT29-N2 cells were imaged in a confocal spinning disk microscope. One complete z-stack was acquired every 20 seconds. Shown is the lateral maximal projection of the imaged cells. The intensity of mucin5-AC·GFP fluorescent emission was color-coded in a look-up table for better visualization. Mucin granules look saturated to better visualize mucin secretion to the extracellular space.

Supplementary Video 3 : Time-lapse of WT and TSPAN8 KO HT29-N2 cells during ATP stimulation. As supplementary video 2 but shown is the maximal top-view projection.

Supplementary Video 4 : Movie of a confocal z-stack of HT29-N2 cells over expressing Tspan8·RFP and immunostained for the NaKATPase. Movie showing a 3D projection of HT29-N2 cells over expressing Tspan-8·RFP and immunostained for the NaKAPTase alpha 1. In the merged image DAPI was used to visualize the cell nucleus.

Supplementary Video 5 : Movie of a confocal z-stack of live HT29-N2 cells expressing Tspan8·GFP. Movie showing a 3D projection of HT29-N2 cells CRISP/Cas genetically-modified to express Tspan8·GFP under endogenous regulation. The plasma membrane was visualized by staining with the lipiphillic die Cellbrite.

Supplementary Video 6 : Movie of a confocal z-stack of live HT29-N2 cells over expressing Tspan8·mScarlet and GFP·Stx2. Movie showing a 3D projection of a stable HT29-N2 cell line over expressing Tspan-8·mScarlet and GFP·Stx2. The creation of the cell lines was done by lentiviral infection.
